# Slower growth, indoor environmental enrichment, or covered veranda access: A comparison of three rearing systems for broiler chicken welfare and behavior

**DOI:** 10.1016/j.psj.2026.107950

**Published:** 2026-10-11

**Authors:** F. Mocz, C. Boulangé, J-P. Moysan, L. Warin, A. Keita, C. Arnould, B. Méda, E. Le Bihan-Duval, M. Guinebretière

**Affiliations:** a Epidemiology, Health and Welfare Unit, Ploufragan-Plouzané-Niort Laboratory, French Agency for Food, Environmental and Occupational Health & Safety (ANSES), Ploufragan, France; b Technical Institute for Poultry (ITAVI), Nouzilly, 37380, France; c Avian Experimental Unit, Ploufragan-Plouzané-Niort Laboratory, French Agency for Food, Environmental and Occupational Health & Safety (ANSES), Ploufragan, France; d INRAE, CNRS, Université de Tours, PRC, Nouzilly, 37380, France; e INRAE, Université de Tours, BOA, Nouzilly, 37380, France

**Keywords:** Poultry, Growth rate, Enrichments, Covered veranda, Behavior

## Abstract

The use of slower-growing chicken strains and environmental enrichments are major levers for improving broiler welfare. This study compared the effects of three rearing systems on the welfare and behavior of broiler chickens: intermediate-growing broilers (Redbro) kept in an unenriched environment (“RDB”), fast-growing broilers (Ross 308) kept in a highly enriched and complex indoor environment (“RoE+”), and fast-growing broilers (Ross 308) having access to a 36-m² covered veranda from D14 (“RoCV”). Each of the four 81-m² pens per treatment housed 1,350 birds until they reached a final body weight of 1.9 kg, corresponding to a final indoor stocking density of 30 kg/m². As they aged, the RoCV chickens increasingly went into the covered verandas until an average of 27% of the birds in the verandas was reached on D32. At the same time, RoE+ broilers perched more and more on the enrichments over time. RoE+ birds consumed more water than RoCV chickens from D24 onward, without any difference in feed consumption. RDB and RoCV birds had fewer leg lesions than RoE+ ones, although the litter quality did not differ between the treatments, but RDB birds had better gait scores than either RoE+ or RoCV broilers. More walking, running, foraging, sparring and frolicking behaviors were observed in the covered verandas than in the indoor pens, where resting behavior was more frequently observed. RoE+ birds dustbathed more often than RDB and RoCV chickens. No difference between treatments was found for mortality. Fewer RoCV broilers reached the different steps of the detour test and approached a novel object than RDB and RoE+ broilers. Overall, the three rearing systems compared influenced different aspects of broiler welfare: RDB treatment was associated with better walking ability and fewer occurrences of footpad dermatitis, RoE+ with more comfort behaviors, and RoCV with more active behaviors and improved footpad condition.

## Introduction

In many livestock productions, animal welfare has become a major concern. However, production in the poultry sector has been intensified to a greater degree than all other livestock species (Bessei, 2018), leading to requests of better rearing conditions by consumers and NGOs through initiatives like the European Chicken Commitment (ECC) concerning the production of broiler chickens (ECC, 2019). The ECC, an initiative by European animal protection organizations, proposes welfare criteria and standards to improve broilers rearing conditions. They include a maximum stocking density of 30 kg/m², the use of slower-growing strains or environmental improvements such as perches and pecking substrates. Indeed, there is a scientific consensus that reducing growth rate, lowering stocking density and providing birds with environmental enrichments induce major welfare improvements (Dixon, 2020; Rayner et al., 2020; Guinebretière et al., 2024; EFSA, 2023).

Since the 1960s, there has been a massive increase in the growth rates of commercial broiler lines. For example, from 1957 to 2005, broiler growth increased by over 400%, with a concurrent 50% reduction in feed conversion ratio (Zuidhof et al., 2014). Usually, broilers with a weight gain above 60 g per day are considered fast-growing, those with a maximum weight gain of 50 g per day are considered intermediate, and those with a maximum growth rate of 45 g per day as slower-growing broilers (EFSA, 2023). Fast-growing strains such as the Ross 308 have been selected to prioritize rapid growth rates, high feed efficiency and large breast muscle sizes (Tallentire et al., 2016). However, this genetic selection focusing primarily on production traits has affected several physiological and behavioral parameters, negatively impacting the welfare of fast-growing broiler chickens. These negative effects include reduced leg health, poorer gait and lower overall physical activity, leading to extended periods spent sitting or lying, which can in turn result in skin lesions (contact dermatitis) due to prolonged contact with wet litter (Hartcher and Lum, 2020; Santos et al., 2022; Riber and Wurtz, 2024). Nevertheless, Riber and Wurtz (2024) indicated that even minor reductions in growth rate can lead to improvements in welfare.

The expression of natural behaviors is more stimulated in alternative systems that provide indoor enrichments than in barren, confined rearing environments (Riber et al., 2018; Mocz et al., 2025). The expression of species-specific behaviors is central to bird welfare. By definition, welfare is not assessed solely by the absence of negative affective states but also by the presence of positive affective experiences arising from highly motivated and rewarding behaviors that generate comfort, pleasure, interest, and a sense of control (Mellor, 2016; Anses, 2018). For example, foraging, dustbathing, and play are highly motivated behaviors associated with positive affective states and positive welfare outcomes (Boissy et al., 2007; Held and Špinka, 2011; Bach et al., 2019; Rayner, 2020; Papageorgiou, 2023). Play is often described as an “opportunity” or “luxury” behavior because it tends to disappear when animals are sick or injured, when resources are limited, or when environmental conditions are perceived as unsafe. As a rewarding and socially contagious activity, play can contribute to animal welfare in both the short and long term (Špinka et al., 2001; Held and Špinka, 2011; Baxter et al., 2019; Papageorgiou, 2023). Pleasurable in the short term, play has long-term functional benefits, allowing the development and acquisition of somatic skills and competencies that help animals adjust to future stressful situations (Held and Špinka, 2011; Mellor, 2015). Some studies have shown that indoor enrichments did not affect the play behavior of fast-growing broilers (Baxter et al., 2019), and could even negatively impact it (Liu et al., 2020). However, indoor environmental enrichments are known to stimulate the expression of other natural behaviors such as perching or foraging, and to reduce emotional reactivity and improve birds’ welfare (Riber et al., 2018; Vasdal et al., 2019; Guinebretiere et al., 2025).

Few studies have examined the behavior of broiler chickens in covered verandas, but available data indicate that such areas stimulate active behaviors in fast-, intermediate- and slow-growing broilers (Bergmann et al., 2017; Mocz et al., 2025). However, the expression of play behaviors has never been studied among broiler in covered verandas. A covered veranda is a non-heated area adjacent to the barn, accessible to the birds via popholes in the side of the barn and partially exposed to outdoor environmental conditions (EFSA, 2023). The covered veranda allows birds to have access to outdoor conditions while reducing their exposure to the risks of disease and predation, as the roof and wire mesh provide a barrier against wild birds, predators and other animals. The covered veranda provides birds with additional space (though only if its surface is not included in the stocking density calculation), the opportunity of choosing between an indoor and outdoor environment and offers them the possibility of accessing daylight and outdoor conditions such as fresh air, sunlight and varying temperatures. In that respect, covered verandas are considered as environmental enrichments, which are defined as an improvement of the environment of captive animals, which increases the behavioral opportunities of the animal and leads to improvements of the biological function (Riber et al., 2018).

In this context, the aim of our study was to compare the effects of three rearing systems on the leg health, performances and behaviors of broiler chickens. Emotional reactivity and cognitive ability were also assessed through a novel object test and a detour test, respectively, as these traits are known to be influenced by the rearing environment. (Riber et al., 2018; Guinebretiere et al., 2025). Each system incorporated different levers (slower growth rate, indoor enrichments, enrichment via an access to a covered veranda) to improve bird welfare: intermediate-growing broilers kept in a limited indoor-enriched environment, fast-growing broilers kept in a highly enriched indoor environment, and fast-growing broilers kept in a limited indoor-enriched environment but with access to a covered veranda.

## Materials and methods

### Ethics statement

All the housing, management and experimental procedures complied with European legislation on the protection of animals used for scientific purposes (EU Directive 2010/63/EU) and on the protection of chickens kept for meat production (Council Directive 2007/43/EC), and were approved by the ANSES ethical committee (D-22-745-1, Opinion no. 2023-07-11-09).

### Housing and experimental design

A total of 10,800 one-day-old Ross 308 broilers (fast-growing strain, 61 g/d, from 33-week-old breeders) and 5,400 one-day-old Redbro broilers (intermediate growth rate, 49 g/d, from 51 to 63-week-old breeders) were provided on the same day in September 2023 (*Galina Perrot* hatchery, La Roche-Jaudy, France) to the ANSES Avian Experimental Unit in Ploufragan, France. The chicks were placed in 12 pens of 81 m² within the same experimental broiler house that has six rooms (two pens per room), without mixing the strains. Redbro chicks were randomly split into two rooms and four pens, and Ross 308 chicks into four rooms and eight pens. Broilers of each strain were placed in different rooms because Ross 308 chickens were slaughtered earlier than Redbro broilers. This arrangement prevented the remaining birds being disturbed after the first treatment batch was sent to the slaughterhouse. As slaughter was planned at a similar final body weight, around 1.9 kg, rearing periods differed between strains according to their different growth rates: 33 days and 40 days for Ross 308 and Redbro broilers, respectively. The targeted indoor stocking density at the end of the rearing period was 30 kg/m² in all treatments.

All rooms had access to natural light through eight windows (90 cm x 30 cm, with shutters closed during night hours) from 08:00 to 19:00. The natural light was supplemented, if needed, by artificial light guaranteeing 30 lux for 18 h per day from 6 days of age (from 06:00 to 00:00). Sawdust litter was provided to birds in each pen following standard management practices (1.2 kg/m² on a concrete floor). All chickens had *ad libitum* access to the feed and water. The same diets (starter: 0-8 days; grower: 9-28 d; finisher-withdrawal: 29 d-slaughter) were used for the two genetic strains and were tailored to the requirements of the most demanding strain (i.e. Ross 308). In each pen, broilers also had access to an automatic round hanging scale (8 cm high, 60 cm diameter).

In this study, three different rearing systems were compared (2 rooms per treatment; Fig. 1):

• **RDB**: 1,350 Redbro broilers per pen reared indoors with one alfalfa bale per pen (20 kg, 30 cm high × 50 cm long × 30 cm wide) and one pecking block composed of minerals (oyster shells) and organic inclusions (extruded linseed, brewer's yeast, extruded wheat and a mixture of aromatic plants) weighing 8 kg (12 cm high × 19 cm long × 19 cm wide), renewed if needed.
• **RoCV**: 1,350 Ross 308 broilers per pen reared with an enriched environment similar to RDBs (i.e. one alfalfa bale and one pecking block, renewed if needed) and an access to a covered veranda of 36 m² (not taken into consideration for the stocking density calculation) from 14 days of age. Each covered veranda had two 24/24 h open popholes. The concrete floor of the covered verandas was covered in straw pellets (2 kg/m², without any fresh addition), and the side looking out onto the outdoor area was made of wire mesh. The roof was equipped with an anti-drip film to prevent condensation and dripping onto the broilers. Windbreaker nets were installed on the entire height of the wired walls to protect the broilers from rain and wind.
• **RoE+**: 1,350 Ross 308 broilers per pen reared indoors with a complex enriched environment including:

- visual markers to help animals locate (colored markings on the walls, on the weighing chains and feed hopper chains);
- one elevated platform (30 cm high, plastic slatted floor) of 2 m² (1 m wide × 2 m long) with access ramps of 1 m² (1 m × 1 m, plastic slatted floor) from both sides (4 m² of platform + ramps in total) with black fringes underneath to create a shaded area under the platform and the ramps (Josse, Montauban-De-Bretagne, France);
- one linear perch (10 cm high × 100 cm long × 10 cm wide), with a mushroom-shaped section (Lubing, Sailly-Sur-La-Lys, France);
- two vertical dividers (50 cm high × 100 cm long × 5 cm wide) (Josse, Montauban-De-Bretagne, France);
- one dust pan (21 cm high × 77 cm long × 87 cm wide) filled with wood dust from D11;
- two perforated dispensers (15 cm high x 8.5 cm wide) filled with grit for pecking, suspended at bird height;
- two alfalfa bales (similar to those in the RDB and RoCV pens), renewed if needed;
- two pecking blocks (similar to those in the RDB and RoCV pens).

**Fig. 1:**
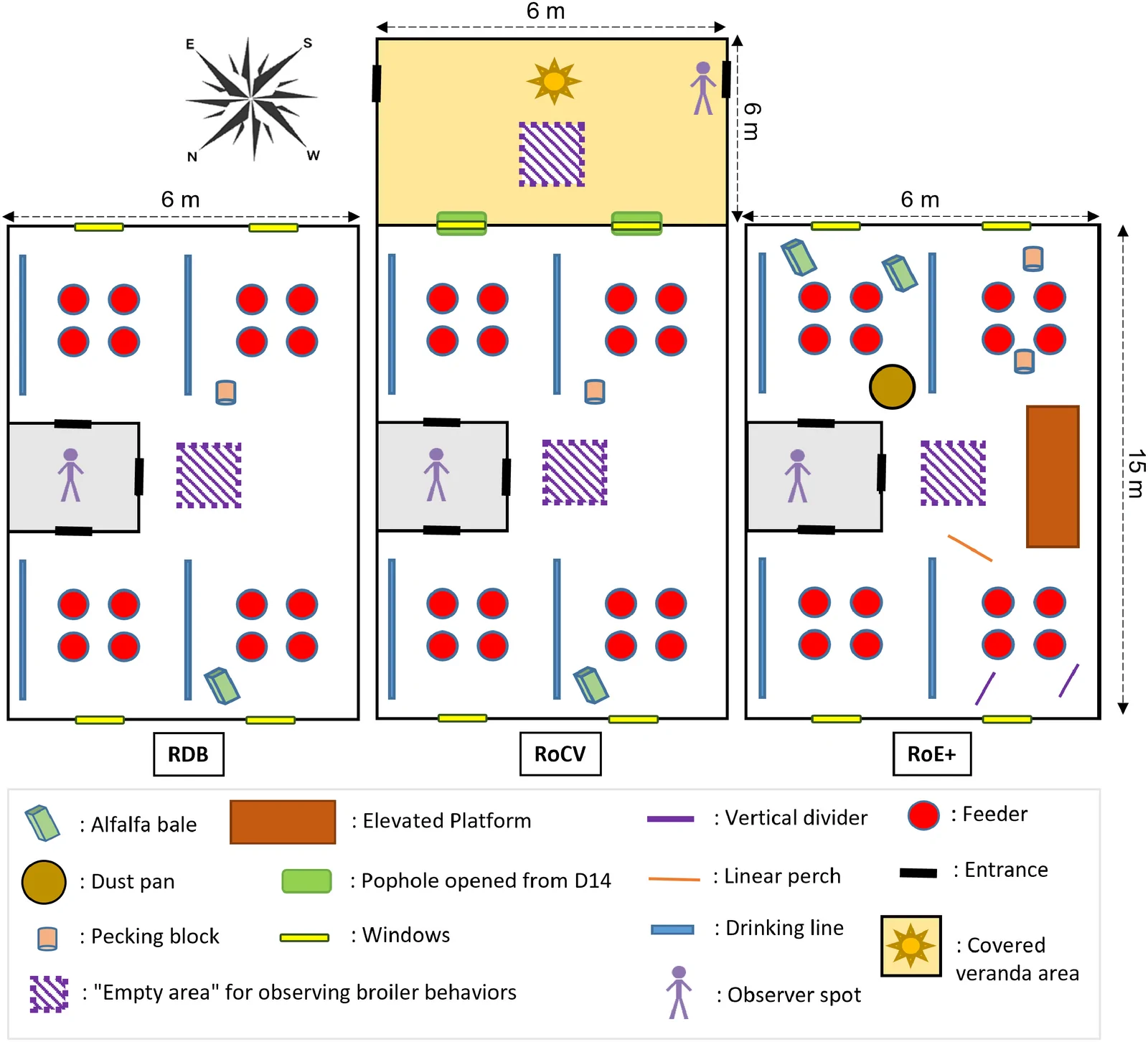
Schematic view of one pen from each experimental treatment. RDB: Redbro broilers reared indoors with a limited enriched environment. RoCV: Ross 308 broilers with access to a covered veranda from 14 days of age. RoE+: Ross 308 broilers reared indoors with a complex enriched environment.

Indoor environmental conditions (temperature and humidity programs) were tailored to the needs of each strain, although the temperature in RoCV rooms was decreased faster than in RoE+ rooms, from 10 days of age, to facilitate birds' adaptation to outdoor conditions ahead of the opening of covered veranda access.

### Data collection

Leg health (footpad dermatitis, hock burns), gait score, and behaviors, which are particularly sensitive to body weight, were assessed both at the same age and at similar body weights in order to avoid confounding age and weight effects when comparing strains with different growth rates (Santos et al., 2022; Guinebretière et al., 2024). Logistical constraints prevented the detour and novel object tests from being conducted on various days for comparison at similar weights.

***Animal performance***. A total of 160 randomly-selected birds per treatment (40 per pen, gender balanced) were manually and individually weighed on D7, D14, D21, D27, D32 for the three treatments and on D38 for RDB broilers. The number of dead broilers (found dead or culled if needed to avoid suffering) was recorded daily, in addition to feed and water consumption for each pen.

***Leg health and gait score.*** On D27 and D32 for the three treatments and on D38 for RDB birds, broilers selected for weighing were also assessed for the presence and severity of footpad dermatitis and hock burns by two trained assessors. Scoring ranged from 0 to 2 points for each criterion (Table 1). After weighing, the first assessor examined both legs and scored the condition of the worst one. The bird was then returned to the pen. On D32 for all treatments and D38 for RDB birds only, the second assessor recorded the gait score while observing the bird moving back to the pen from behind, gently encouraging it to walk with a stick if needed.

**Table 1:** Scoring of footpad dermatitis, hock burns and gait (Guinebretière et al., 2024).

	Score 0	Score 1	Score 2
Footpad dermatitis	None or slight, very superficial lesions, slight discoloration over a limited area, slight hyperkeratosis or scarred skin	Moderate to severe discoloration, superficial lesion, darkened papillae	Severe dermatitis ulcers or scabs of significant size, signs of bleeding or severely swollen foot pads
Hock burns			
Gait score	Normal gait, agile, with or without imbalance	Broiler walks more than 1.50 m but has difficulty in walking; limps	Broiler walks less than 1.50 m, and/or is severely lame, preventing movement

***Litter quality.*** On D27, the litter quality of each room was assessed by a single assessor in the same three areas in each pen (in the middle of the pen, under the drinkers and between feeders), giving three scores per room. The friability scoring system of the ClassyFarm protocol (Table 2; Vinco et al., 2018, 2023) was chosen after comparison of its validity, inter-assessor reliability and intra-assessor repeatability with other litter scoring systems (Mocz et al., 2024).

**Table 2:** Description of the friability scoring system of the ClassyFarm protocol (Vinco et al., 2018, 2023).

Score	Friability description
1	Completely caked
2	80-90% of area caked
3	70-80% of area caked
4	60-70% of area caked
5	50-60% of area caked
6	40% of area caked
7	30% of area caked
8	10% of area caked
9	Friable litter, small caked areas
10	Friable litter, no caked areas

***Behaviors.*** The behaviors of the broilers in each treatment group were recorded on D15, D18, D20, D22, D26 and D29 for the RDB treatment over a 3-min period twice a day, in order to compare the treatments at the same age (from D18 to D26) and similar body weight (675 g = Ross 308 on D18 vs. RDB on D20; 800 g = Ross 308 on D20 vs. RDB on D22; 950 g = Ross 308 on D22 vs. RDB on D26; and 1,200 g = Ross 308 on D26 vs. RDB on D29). This 3-min period consisted of direct *ad libitum* observations of a 4 m² ‘empty’ observation area (i.e. away from feeders, drinkers and enrichments; Fig. 1) in each pen and in each covered veranda for the RoCV treatment, between 09:00 and 17:00. Following a 1-min period for the birds to get used to the observers’ presence, two observers carried out the observations simultaneously (the behaviors recorded were split between the observers) based on the ethogram presented in Table 3 (adapted from Lourenco da Silva et al., 2021; Baxter et al., 2019; Mocz et al., 2025; Guinebretiere et al., 2025). The first observer noted the walking, running and lying behaviors; the second observer recorded the foraging, dustbathing and playing behaviors. Every occurrence of each behavior was noted without individual identification (all occurrences sampling). If a bird interrupted a behavior for more than 5 seconds, a new occurrence was counted when the behavior was resumed. As a result, multiple behavioral events could be attributed to a single bird within the 3-min observation period, either for the same behavior or for different ones. The total number of broilers, including birds lying down (i.e. lying on the litter with its head either resting on the ground or erect, pecking the litter or not, whether eyes were open or shut), was noted before and after each 3 min observation period by scan sampling.

**Table 3:** Experimental ethogram used for the assessment of broiler behaviors (adapted from Baxter et al., 2019; Lourenco da Silva et al., 2021; Mocz et al., 2025; Guinebretiere et al., 2025).

Behavior	Definition
Walking	Chicken moves more than 3 steps at a slow pace.
Running	Chicken moves more than 3 steps at a fast pace.
Foraging	Chicken stands in an upright position with both feet on the ground, uses both feet alternatively to scratch at the ground, and/ or lowers its head from time to time to peck at or move litter material.
Dustbathing	Chicken pecks and scratches at the litter material, then crouches down in the substrate, and follows an organized sequence of behavioral patterns (crouching, stirring the litter and shaking).
Sparring game	Brief fighting behavior with no aggression or injurious contact: jumping with slight kicking motions that make little or no contact with the receiver; stand-offs (threats) in which birds will face up to one another briefly, stepping close and raising their necks to stand practically beak-to-beak; raising feathers around the neck, usually during a stand-off; stand-off with wing-flapping; stand-off with light pecks at the neck, head or beak of the receiving bird.
Frolicking	Spontaneous and rapid running and/or jumping and wing-flapping, often with rapid changes in direction.

***Covered veranda use.*** The number of RoCV chickens observed in each covered veranda was counted by a single observer from one to three times a day (morning and/or afternoon) on D15, from D18 to D22, from D25 to D27, on D29 and finally on D32. The time of day (hour), as well as the temperature and humidity levels in the covered veranda, were noted for each of these counting periods.

***Use of enrichments in RoE+ pens.*** The number of RoE+ chickens perched on the platform and its ramps, on the alfalfa bales, on the linear perch, or seen in the dust pan was noted by a single observer directly in the pen from one to two times a day on D1, from D4 to D8, from D11 to D15, from D18 to D22 and finally from D25 to D27.

***Detour test.*** On D28 and D29, the chickens’ spatial problem-solving ability was assessed using the detour test (Wichman et al., 2009; Guinebretiere et al., 2025). To reduce the bias of different days/ages and the behavioral differences between morning and afternoon, the same number of chickens from each treatment group was tested in each half-day (15 birds per treatment per half-day) over the two test days. A total of 60 birds per treatment (15 per pen) were successively placed in a test arena (100 cm long × 100 cm wide × 60 cm high) behind a U-shaped mesh fence (45 cm long x 45 cm wide x 60 cm high). From there, each bird could see its conspecifics in the rearing pen through the mesh but could only reach them by circumventing the fence. The test arena was placed on one side of the rearing pen to maintain identical floor and ambient conditions and to limit the stress of a novel environment. The bird being tested was placed in the closed U-shaped mesh fence then given one minute for adaptation before the panel closing the back of the U was removed and the test began. To increase the bird’s motivation to reach the goal, dried mealworms were placed in a cup within the U-shaped area close to the tested bird, and in an identical cup in the arrival area, both visible from the starting point. The following variables were recorded: success in exiting the U-shaped area (point 1, Fig. 2), success in going around the U-shaped fence (point 2), success in going beyond the U-shaped fence (point 3), success in reaching the other broilers and the worms (point 4), along with the corresponding latencies. The test lasted ended as soon as the bird successfully reached its conspecifics, or a maximum of 5 minutes. In the event of failure to achieve any step of the test, the maximum latency value of 5 minutes was noted.

**Fig. 2:**
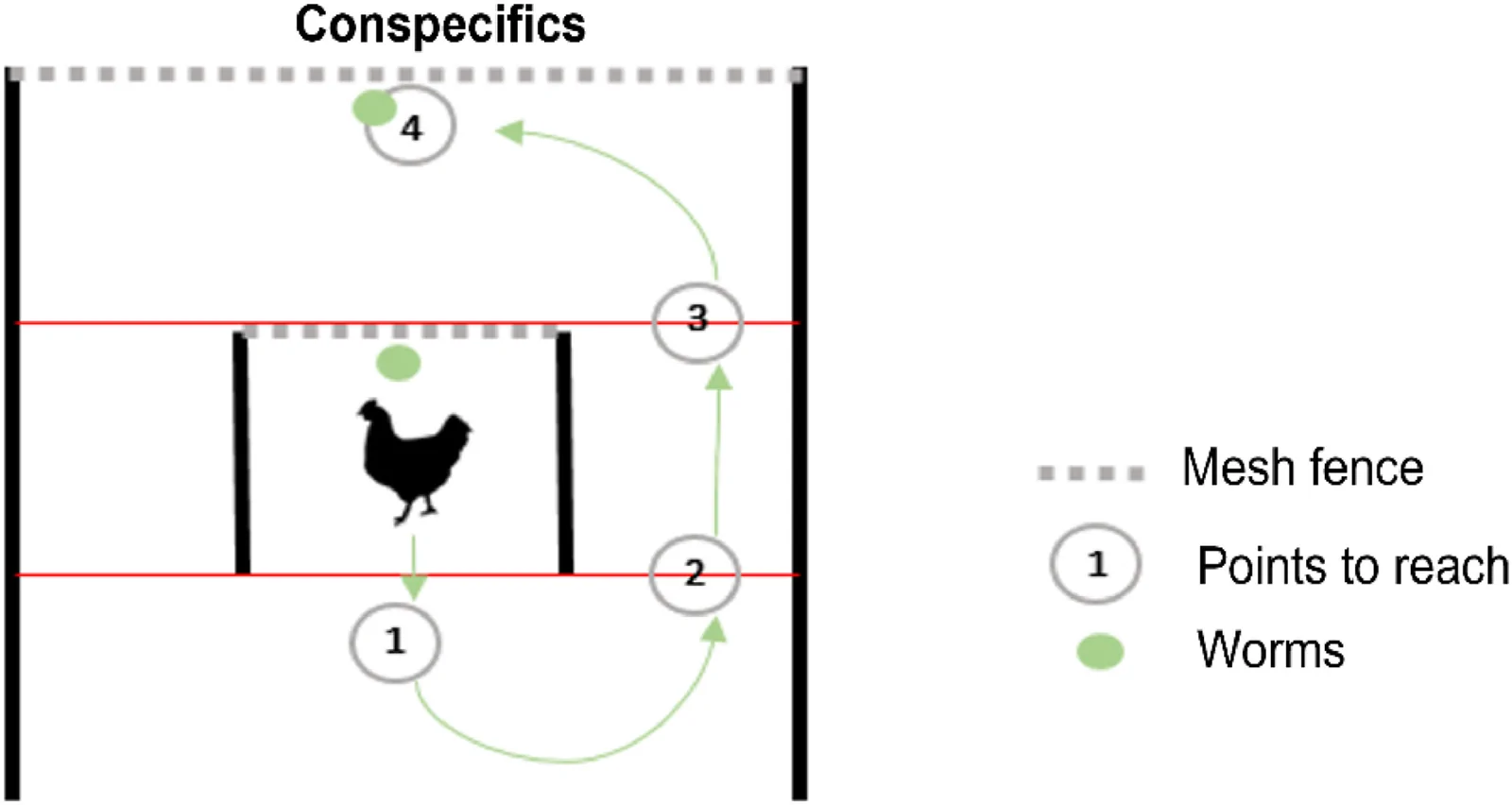
Schematic description of the steps involved in the detour test.

***Novel object test.*** Reactivity to a novel object was assessed in each pen by a single observer between 14:00 and 17:30 on D13 and D27. The same object, a tower of stacked multi-colored plastic blocks measuring about 40 cm in height and 18 cm in width, was used on both test days. The 10-minute test was performed in the rearing pen and involved the entire group so as to avoid the stress of socially isolating the birds. The observer entered the pen, put the novel object in a free area (far from feeders, drinkers and enrichments), then moved 6 m away to the observer’s spot, outside the pen (see Fig. 1). The time taken (latency) for the first broiler to walk within 30 cm of the novel object and the time taken for the first broiler to peck the novel object were recorded. In addition, the number of broilers within 30 cm of the object and the number of broilers pecking the object were recorded every 30 seconds.

### Statistical analysis

Data were pre-processed and analyzed using R software v. 4.0.3 (R Core Team, 2023). Several statistical models were tested for each dataset, incorporating different variables as fixed or random effects. The type of model (distribution family and random-effects structure) was chosen a priori for each response variable based on its measurement scale and sampling design, and checked against the relevant model assumptions. Within each model type, we compared nested models differing in their fixed-effects structure (inclusion/exclusion of explanatory variables and their interactions) using AIC (Akaike Information Criterion) and BIC (Bayesian Information Criterion), and retained the model offering the best trade-off between fit and parsimony. The experimental unit was the pen for all analyses except for body weights, leg health and detour test where the statistical unit was the individual bird. However, water consumption was measured at the room level due to technical limitations preventing accurate measurement at the pen level. Consequently, analyses of water consumption were conducted using the room as the experimental unit.

***Growth performance, feed and water consumption.*** Body weight, mortality, daily feed and water consumption were analyzed using mixed linear models. These variables are continuous and were repeatedly measured over time within the same pens, so pen (or room, for water consumption) was included as a random effect to account for this non-independence. Body weight, daily feed and water consumption models included pen (or room for water consumption) as a random effect, with treatment, age and the interaction between both as fixed effects. The mortality model included pen as a random effect, with treatment and age as fixed effects.

***Use of environmental enrichments.*** Because these outcomes were counts, overdispersed relative to a Poisson distribution, generalized linear mixed models (GLMM) with negative binomial distribution were used to analyze number of RoCV broilers observed in covered verandas and the use of platform and alfalfa bales by RoE+ broilers. For covered veranda use (RoCV pens), the model included age, hour, temperature and interactions between age and hour and between temperature and humidity as fixed effects, with pen as a random effect. The models analyzing use of indoor enrichments (RoE+ pens) included age as a fixed effect and pen as a random effect. To study perching preferences of RoE+ broilers on the platforms, the model included effects of the platform area (top or ramps) and age together with their interaction as fixed effects, and pen as a random effect. There was insufficient variability in the data on the use of the dust pan and linear perch to ensure model quality, so only descriptive analyses were retained. To visualize temporal trends in (i) the percentage of RoCV broilers observed in covered verandas and (ii) the use of enrichments by RoE+ broilers, smoothing curves were fitted using locally estimated scatterplot smoothing (LOESS). The smoothing parameter was set to the default value in the ggplot2 package.

The correlation between temperature and humidity recorded in the covered verandas was analyzed using a Pearson correlation test.

***Behaviors.*** To quantify broilers’ behaviors, standardized occurrence rates (SORs) were calculated by dividing the total occurrences of each behavior (Table 3) observed during the 3-min observation period by the number of birds present in the 4 m² observation area during those same 3-min of observation (averaging the number of birds counted in the area before and after the 3-min of observation), and then multiplying by 100 to express the rate as occurrences per 100 broilers. In addition, the percentage of broilers lying down in the area was noted before and after the 3-min of observation. Mixed linear models were used to analyze the behaviors. These models included the treatment, with a distinction between behaviors observed indoors or in the covered veranda for the RoCV treatment (RoCV-I and RoCV-CV, respectively), as a previous study (Mocz et al., 2025) showed clear contrasts between behaviors expressed in the covered verandas and those expressed inside the poultry house. Different models were used depending on the type of comparison. For comparison at the same age and similar weights, mixed linear models were used that included treatment as a fixed effect and pen as a random effect. When the pen-level variance component was estimated near zero (singular fit), indicating no meaningful contribution of the random effect for that behavior, a simpler linear model without the random effect was applied instead.

***Novel object test.*** As for the enrichment-use models above, approach and pecking behaviors were count data, so a GLMM with negative binomial distribution analyzed approach and pecking behaviors, with treatment, age, time point within the test (0–10 min, sampled every 30 s) and treatment × age interaction as fixed effects and pen as a random effect. Latencies were compared using Kruskal-Wallis tests.

***Non-normal and categorical outcomes.*** In the absence of residual normality, even after logarithmic or square root transformation, non-parametric tests were used for comparison across treatments: Kruskal-Wallis tests for litter quality and detour test latencies, with Dunn-Bonferroni post hoc comparisons. Leg health scores (gait score, footpad dermatitis and hock burn) were treated as categorical, and for each score category, chi-square or Fisher's exact tests (depending on expected cell counts) compared the proportion of affected animals between treatments. The same approach was used to compare the percentages of birds succeeding in reaching each point (see Fig. 2) and eating the worms in the detour test’s starting U-shape fence.

***Comparisons at same age and body weight.*** As indicated before, metrics on leg health and behaviors were compared both at the same age and at similar body weights. For leg health indicators (footpad dermatitis, hock burns and gait score), the RDB treatment group on D38 was compared with both Ross 308 treatments (RoE+ and RoCV) on D32, corresponding to a similar body weight (approximately 1,870 g). Observed behaviors were compared at similar body weights: approximately 675 g (i.e. Ross 308 treatments on D18 vs. RDB on D20); 800 g (i.e. Ross 308 treatments on D20 vs. RDB on D22); 950 g (i.e. Ross 308 treatments on D22 vs. RDB on D26); and 1,200 g (i.e. Ross 308 treatments on D26 vs. RDB on D29).

For all analyses, significant interactions were followed by post-hoc analyses of estimated marginal means with a Tukey adjustment. Differences were considered significant when *P* ≤ 0.05.

## Results

### Animal performance

***Body weight.*** Significant effects of the treatment, the age, and the treatment × age interaction were found on the body weights (BW) (*P* < 0.001 in all cases). There was a significant effect of the strain on BW, the RDB broilers being lighter than both Ross 308 treatments from D14 until slaughter (*P* < 0.001; Table 4). Furthermore, RoE+ broilers were significantly heavier than RoCV broilers on D27 (+ 4.5% in average, *P* = 0.004).

**Table 4:** Mean weights (g) (±standard deviations) of broilers from each treatment on D7, D14, D21, D27, D32 and, additionally for RDB treatment only, on D38. RDB: Redbro broilers reared indoors with a limited enriched environment. RoCV: Ross 308 broilers with access to a covered veranda from 14 days of age. RoE+: Ross 308 broilers reared indoors with a complex enriched environment.

	RDB	RoE+	RoCV	Significance of treatment effect (*P*)
D7	164 (19)^a^	188 (68)^a^	173 (21)^a^	0.45
D14	386 (50)^a^	458 (53)^b^	462 (54)^b^	<0.001
D21	740 (124)^a^	913 (110)^b^	924 (112)^b^	<0.001
D27	1106 (164)^a^	1464 (197)^b^	1401 (213)^c^	<0.001
D32	1537 (186)^a^	1873 (267)^b^	1847 (275)^b^	<0.001
D38	1899 (302)			

Different superscript letters within a row indicate significant differences between treatments (P < 0.05), as determined by post hoc tests following the significant Treatment × Age interaction.

***Mortality.*** There was no significant effect of treatment on total mortality (*P* = 0.17). The total mortality was 2.4% (standard deviation: 0.8%) for the RDB treatment, 3.5% (0.8%) for the RoE+ and 3.3% (1.3%) for the RoCV.

***Feed and water consumption.*** To reach the same final body weight of 1.9 kg, RDB broilers consumed a total of 3,294 g (standard deviation: 104) of feed and 6,379 mL (standard deviation: 157) of water per bird; RoCV broilers consumed 2,774 g (104) of feed and 5,330 mL (87) of water per bird, and RoE+ broilers consumed 2892 g (37) of feed and 5,587 mL (43) of water per bird.

On a daily basis, there was no effect of the treatment (*P* > 0.05) but a significant effect of the age and the interaction between treatment and age for both feed and water consumption (*P* < 0.001).

On a daily basis, RDB broilers consumed less feed than RoE+ from D8 onward (*P* ≤ 0.02 from D8) and less feed than RoCV from D9 onward (*P* ≤ 0.04 from D9) (Fig. 3). There was no significant difference in feed consumption between RoE+ and RoCV (*P* > 0.05).

**Fig. 3:**
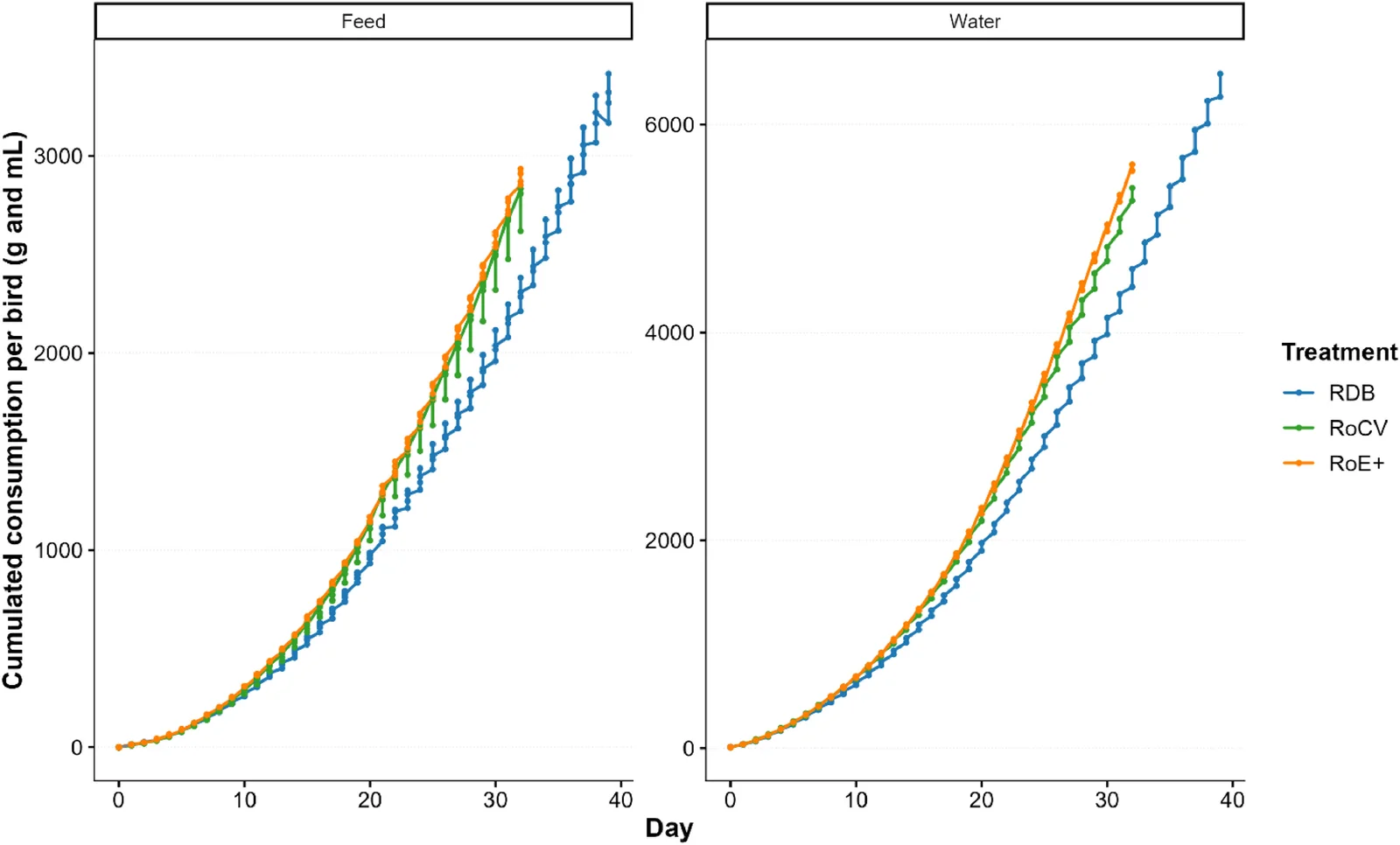
Cumulated feed (g) and water (mL) consumption per bird over the rearing period per treatment. RDB: Redbro broilers reared indoors with a limited enriched environment. RoCV: Ross 308 broilers with access to a covered veranda from 14 days of age. RoE+: Ross 308 broilers reared indoors with a complex enriched.

Regarding daily water intake, RDB broilers drank less water than RoE+ from D9 onward (*P* = 0.03 on D9, and *P* < 0.03 afterwards) and less water than RoCV from D10 onward (*P* = 0.04 on D10, and *P* < 0.04 afterwards) (Fig. 3). RoE+ broilers drank more water than RoCV broilers from D24 onward (***P*** = 0.05 on D24, and *P* < 0.05 afterwards).

### Leg health and gait score

Very few score 2 were found, whether for footpad dermatitis (FPD, 3 out of 160 broilers in the RoE+ treatment on D32) or gait (GS, 1 out of 160 broilers in the RoE+ treatment and 1 out of 160 in the RoCV treatment on D32). No hock burns (HB) were scored 2.

***Footpad dermatitis.*** The effect of treatment was significant at D27, D32, and at a similar BW (1,870 g) (*P* < 0.001; Table 5). RoE+ broilers had more FPD than the other two treatments at all three comparisons. RoCV broilers had more FPD than RDB broilers on D32, but not on D27 or at a similar BW.

**Table 5:** Percentages of broilers that were scored 0 (no disorders) for footpad dermatitis (FPD), hock burns (HB) and gait (GS) on D27 and D32 in each treatment, and on D38 for the RDB treatment only. Treatments were compared at the same age and both strains (Redbro vs. Ross 308) were compared at the same weight (around 1,870 g): Ross 308 on D32 vs. Redbro on D38. RDB: Redbro broilers reared indoors with a limited enriched environment. RoCV: Ross 308 broilers with access to a covered veranda from 14 days of age. RoE+: Ross 308 broilers reared indoors with a complex enriched environment.

Indicator	Age and Body weight	RDB	RoE+	RoCV	Significance of treatment effect (*P*)
FPD	*D27*	100.0^a^	90.0^b^	97.5^a^	<0.001
	*D32*	95.6^a^	71.9^b^	88.7^c^	<0.001
	*D38*	93.1			
	*1,870 g*	93.1^a^	71.9^b^	88.7^a^	<0.001
HB	*D27*	95.6^a^	91.2^a^	90.6^a^	0.18
	*D32*	80.0^a^	68.7^b^	83.1^a^	0.005
	*D38*	43.7			
	*1,870 g*	43.7^c^	68.7^b^	83.1^a^	<0.001
GS	*D32*	100.0^a^	83.1^b^	90.0^b^	<0.001
	*D38*	95.0			
	*1,870 g*	95.0^a^	83.1^b^	90.0^ab^	0.002

Different superscript letters within a row indicate significant differences between treatments (pairwise Chi-2 or Fisher’s exact test, P < 0.05).

***Hock burns.*** No significant treatment effect was found on D27 (*P* = 0.18). On D32, the treatment effect was significant (*P* = 0.005), with RDB and RoCV broilers having fewer hock burns than RoE+; RDB and RoCV did not differ from each other. At a similar BW, the treatment effect was again significant (*P* < 0.001), with RDB broilers having more hock burns than RoE+ and RoCV broilers.

***Gait.*** There was a significant treatment effect on D32 (*P* < 0.001), with more RDB broilers having a normal gait (score 0) than both RoE+ and RoCV broilers. At a similar BW, the treatment effect remained significant (*P* = 0.002): RDB broilers still had better gait than RoE+ broilers, while RoCV gait scores were intermediate and did not differ significantly from the other two treatments (Table 5).

### Litter quality

The mean Classyfarm Friability litter scores (with standard deviation) on D27 were 4.7 (2.4) for RDB, 4.5 (3.1) for RoE+, and 4.7 (3.2) for RoCV, respectively, without any significant effect of the treatment (*P* = 0.45).

### Behaviors

***Comparison of indoor behaviors.*** There was no significant difference (*P* > 0.05) between treatments for indoor observations (RDB, RoE+ and RoCV-I) of walking, running, foraging, sparring game and frolicking behaviors, whether among all days of observation (Table 6) or when both strains were compared at a similar BW of 1,200 g (Table 7) or 675 g, 800 g, and 950 g (see Table A1 in Supplementary Data).

**Table 6:** Mean (± standard deviation) of the standardized occurrence rates for walking, running, foraging, dustbathing, sparring and frolicking behaviors, and percentage of birds lying down in RDB, RoE+, RoCV indoor (RoVC-I) and RoCV in the covered veranda (RoCV-CV). Observations were conducted on D15, D18, D20, D22, and D26; the data presented in this table are the means for each age across the four pens of each treatment. RDB: Redbro broilers reared indoors with a limited enriched environment. RoCV: Ross 308 broilers with access to a covered veranda from 14 days of age. RoE+: Ross 308 broilers reared indoors with a complex enriched environment.

Behavior	Treatment				Significance of treatment effect (*P*)
	RDB	RoE+	RoCV-I	RoCV-CV	
Walking^1^	44.4 (25.3)^b^	33.5 (28.8)^b^	49.2 (53.1)^b^	398.7 (256.2)^a^	<0.001
Running^1^	7.6 (11.6)^b^	3.1 (6.2)^b^	16.2 (35.1)^b^	96.8 (137.5)^a^	<0.001
Foraging^1^	10.4 (12.8)^b^	11.6 (9.8)^b^	28.9 (50.3)^b^	373.9 (197.0)^a^	<0.001
Dustbathing^1^	12.2 (21.6)^bc^	37.6 (36.5)^a^	18.8 (28.6)^b^	0.6 (4.1)^c^	<0.001
Sparring games^1^	3.2 (5.9)^b^	1.6 (3.7)^b^	16.3 (33.6)^b^	162.7 (105.2)^a^	<0.001
Frolicking^1^	15.7 (19.1)^b^	10.0 (9.2)^b^	35.7 (66.3)^b^	366.5 (225.2)^a^	<0.001
Percentage of birds lying down	81.6 (18.5)^b^	93.8 (7.7)^a^	87.3 (20.9)^ab^	23.5 (24.3)^c^	<0.001

1 number of occurrences of behavior per 100 broilers per 3-min observation period Different superscript letters within a row indicate significant differences between treatments (P < 0.05), as determined by post hoc tests.

**Table 7:** Mean (± standard deviation) of the standardized occurrence rates for walking, running, foraging, dustbathing, sparring and frolicking behaviors, and percentage of birds lying down in RDB, RoE+, RoCV indoors (RoVC-I), and RoCV in the covered veranda (RoCV-CV) and comparison of both strains at a similar body weight of 1,200 g (= Ross 308 treatments on D26 vs. RDB on D29). RDB: Redbro broilers reared indoors with a limited enriched environment. RoCV: Ross 308 broilers with access to a covered veranda from 14 days of age. RoE+: Ross 308 broilers reared indoors with a complex enriched environment.

Behavior	Treatment				Significance of treatment effect (*P*)
	RDB	RoE+	RoCV-I	RoCV-CV	
Walking^1^	20.4 (5.2)^b^	23.8 (11.7)^b^	30.1 (16.6)^b^	306.5 (102.3)^a^	**<0.001**
Running^1^	5.0 (3.4)^b^	0.2 (0.5)^b^	2.5 (3)^b^	53.2 (30.3)^a^	**<0.001**
Foraging^1^	1.7 (2)^b^	4.5 (4.6)^b^	3.4 (3)^b^	209.0 (23.8)^a^	**<0.001**
Dustbathing^1^	0.2 (0.5)^b^	15.4 (14.2)^a^	4.1 (6.8)^b^	0.0 (0.0)^b^	**0.001**
Sparring games^1^	1.1 (1.1)^b^	0.3 (0.9)^b^	0.2 (0.6)^b^	80.9 (39.7)^a^	**<0.001**
Frolicking^1^	4.3 (5.7)^b^	3.3 (2.3)^b^	6.3 (4.5)^b^	240.9 (138.9)^a^	**<0.001**
Percentage of birds lying down	72.6 (22.4)^a^	94.4 (24)^a^	89.6 (6.4)^a^	24.7 (19.3)^b^	**<0.001**

1 number of occurrences of behavior per 100 broilers per 3-min observation period Different superscript letters within a row indicate significant differences between treatments (P < 0.05), as determined by post hoc tests.

Across all observation days, the proportion of birds observed lying down indoors was significantly higher in RoE+ broilers than in RDB broilers (+12% in average; *P* = 0.01; Table 6). No other significant difference was detected between others treatments. Such difference was not anymore present comparing at similar BW.

Across all observation days and at a similar BW of 1,200 g, RoE+ broilers dustbathed twice more than RoCV-I and three times as much as RDB broilers (*P* ≤ 0.001; Table 6, Table 7), but no difference was found between RDB and RoCV-I (*P* > 0.05).

***Comparison between indoors and covered verandas.*** Across all observation days, there were more walking, running, foraging, sparring game and frolicking behaviors and fewer lying behaviors observed in RoCV-CV broilers than in RoCV-I, RDB, and RoE+ broilers (*P* < 0.001; Table 6). The same results were observed when the comparisons were made at a similar BW (Tables 7 and A1). Across all days of observation, RoCV-CV broilers dustbathed less than RoCV-I and RoE+ (*P* = 0.01 and *P* < 0.001, respectively), but no significant difference was found in comparison with RDB broilers (*P* = 0.20; Table 6). At a similar BW of 1,200 g, there was no difference in dustbathing behavior between RoCV-CV broilers and RDB or RoCV-I broilers, while RoE+ still exhibited this behavior more frequently (Tables 7 and A1).

### Covered veranda use by RoCV broilers

As broilers aged, they were increasingly present in the covered verandas (*P* < 0.001). The lowest number of RoCV broilers observed in the 36 m² covered veranda was recorded on D15, with an average (4 pens) of 22.5 broilers (standard deviation: 18.6), which represents 1.7% of the total number of broilers in the pen, while the highest number was recorded on D32, with an average of 366.25 broilers (133.4), which is approximately 27% of the total number of broilers of the pen.

The interaction between age and time of day tended to have a significant effect on the use of the covered veranda (*P* = 0.06). While the number of RoCV broilers observed in the covered verandas appeared to be only slightly affected by the time of day before D26, after D26 more birds were observed in the covered verandas in the afternoon than in the morning (Fig. 4), with two peak values at 11:30 (316 broilers, or 23% of the pen’s broilers) and at 13:40 (445 broilers, or 33% of the pen’s broilers). However, time of day (*P* = 0.35), temperature (*P* = 0.45) and humidity (*P* = 0.39) alone did not affect the number of RoCV broilers observed in the covered verandas. There was, however, a significant effect of the interaction between temperature and humidity recorded in the covered verandas on the number of RoCV broilers observed (*P* < 0.001). More RoCV broilers were observed in the covered verandas when the temperature was moderate (between 18°C and 25°C), particularly when the humidity was above 73% (Fig. 5). Conversely, the lowest number of broilers in the covered verandas was observed when the temperature exceeded 25°C and humidity was below 57%.

**Fig. 4:**
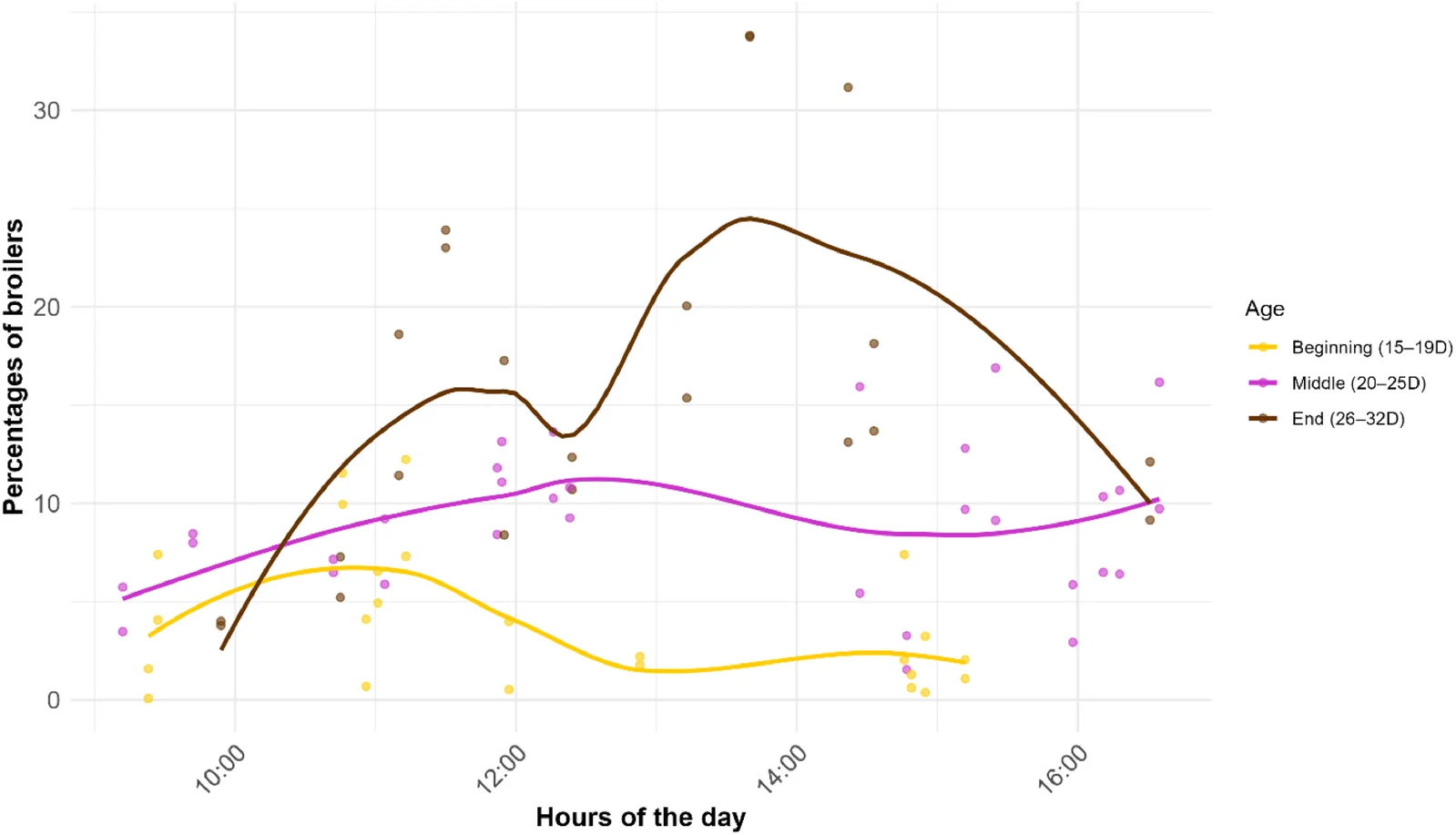
Hourly variation in the percentage of Ross 308 broilers in the pen observed in each covered veranda (RoCV treatment) by age category: 15 to 19 days of age (beginning), 20 to 25 days of age (middle) and 26 to 32 days of age (end). Smoothed trend lines were added using locally estimated scatterplot smoothing (LOESS) to illustrate the general pattern of broiler percentages over the day for each age category.

**Fig. 5:**
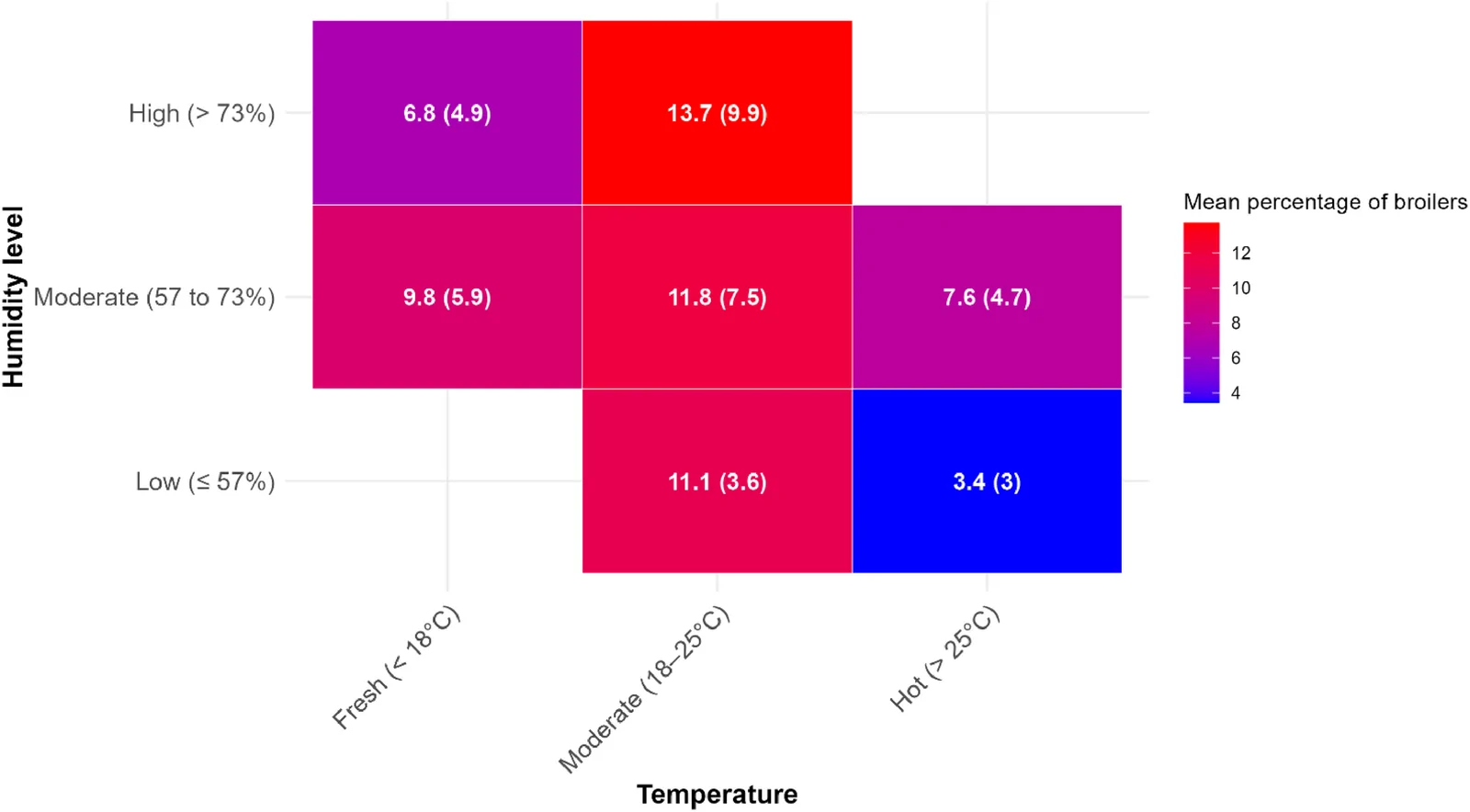
Heatmap on the mean (± standard deviation) percentage of the Ross 308 broilers in the pen (RoCV treatment) according to the significant interaction (p < 0.001) between temperature and humidity level recorded in the covered verandas. Humidity was divided into three categories (low, moderate, high) based on terciles of the observed humidity distribution.

### Enrichments use by RoE+ broilers

As RoE+ broilers aged, they increasingly perched on the top of the platform (*P* < 0.001), on its ramps (*P* = 0.04) and on the alfalfa bales (*P* < 0.001). RoE+ broilers perched more on the top of the platform than on the ramps from D9 (*P* = 0.01, Fig. 6). Broilers were observed perching on the alfalfa bales from D11, and the number of birds perching on both bales reached a maximum of 2.5 (standard deviation: 1.9), which is 0.2% of broilers in the pen, on D19. RoE+ broilers began using the linear perch on D7, reaching a maximum of 3.5 (2.4) birds, or 0.3% of broilers in the pen, on D25. They started using the dust pan on D12, with a maximum of four birds recorded on D25 (0.8) and D26 (1.8), which is 0.4% of broilers in the pen.

**Fig. 6:**
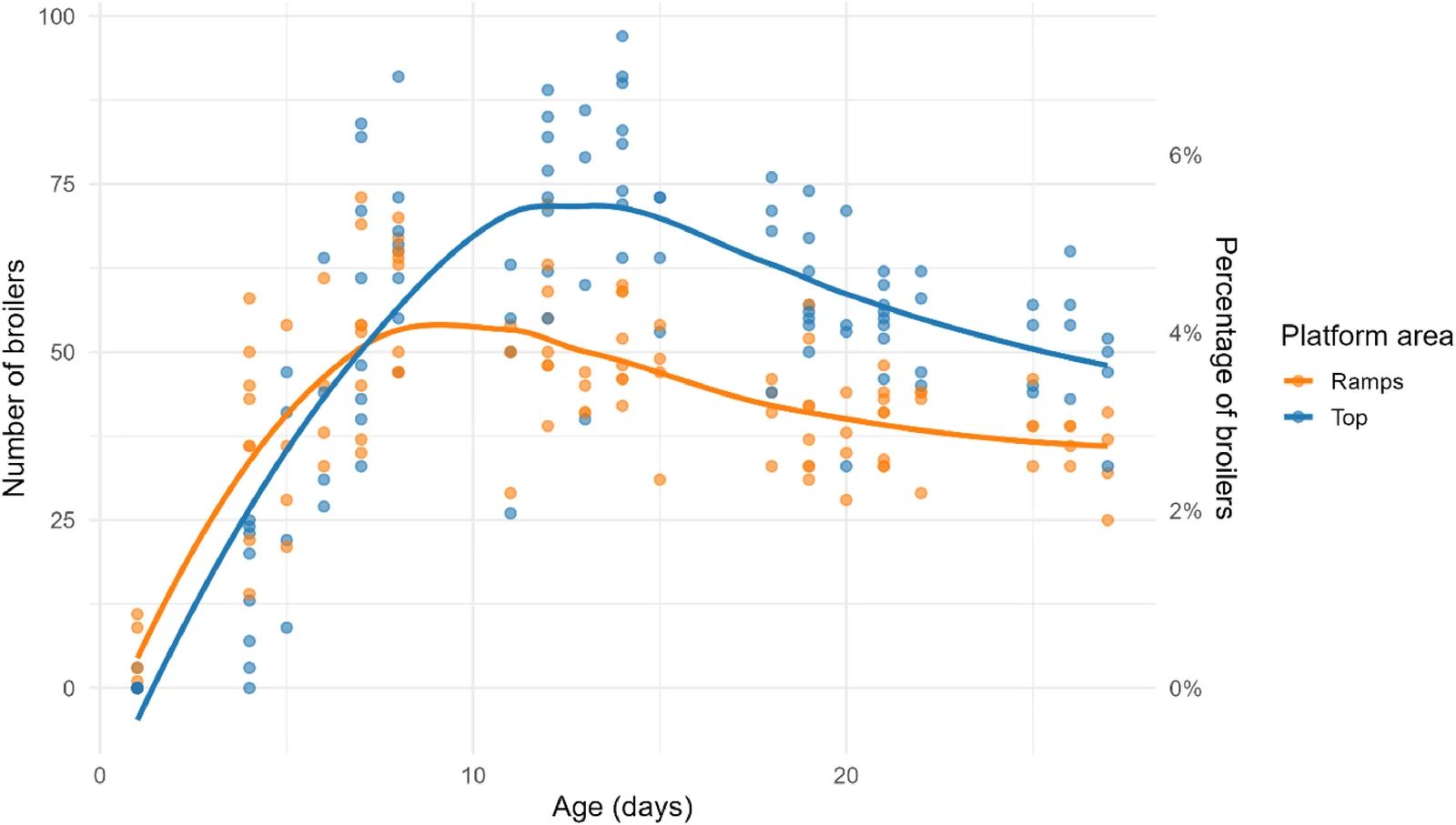
Number and percentages of Ross 308 broilers from the pen reared indoors with a complex enriched environment (RoE+ treatment) perched on the 2 m² top of the platform (blue) or on the 2 m² ramps (orange) over time in days. Smoothed trend lines were added using locally estimated scatterplot smoothing (LOESS) to illustrate the general pattern of broiler numbers over the days.

### Detour test

More RDB broilers succeeded in exiting the U-shaped area (point 1, Fig. 2; *P* = 0.002; Table 8) and did so with a lower latency than RoE+ and RoCV broilers, which did not differ from each other. Moreover, more RDB broilers succeeded, and with a lower latency, in going around the U-shaped fence (point 2) and going beyond it (point 3) than RoCV broilers (respectively *P* = 0.002 and *P* = 0.008), while RoE+ did not differ from the other two treatment groups on these points. There were no significant differences among the three treatments in successfully completing the detour test by reaching their conspecifics (point 4; *P* = 0.15). At the beginning of the test, the treatment did not influence the number of broilers that ate the worms in the U-shaped mesh fence: 61% of RDB broilers, 58% of RoE+ broilers and 45% of RoCV broilers (*P* = 0.15). When considering only the broilers that ate the worms at the start of the test, there was again no significant difference in success in reaching their conspecifics (at point 4) between the various treatments (24% of RDB, 26% of RoE+ and 18% of RoCV broilers; *P* = 0.78).

**Table 8:** Percentages of success and mean latencies in seconds (± standard deviation) to reach each step of the detour test (points, see Fig. 2) for 60 birds per treatment on D28 and D29. RDB: Redbro broilers reared indoors with a limited enriched environment. RoCV: Ross 308 broilers with access to a covered veranda from 14 days of age. RoE+: Ross 308 broilers reared indoors with a complex enriched environment.

Variables		Treatments			Significance of effects (*P*)
		RDB	RoE+	RoCV	
Exiting the U-shaped area (point 1)	*Percentage of success*	68.3^a^	46.6^b^	36.6^b^	0.002
	*Latency*	180 (99)^a^	232 (94)^b^	248 (82)^b^	<0.001
Going arround the U-shaped fence (point 2)	*Percentage of success*	46.6^a^	31.6^ab^	16.6^b^	0.002
	*Latency*	241 (84)^a^	259 (79)^ab^	281 (47)^b^	0.002
Going beyond the U-shaped fence (point 3)	*Percentage of success*	38.3^a^	26.6^ab^	13.3^b^	0.008
	*Latency*	250 (82)^a^	269 (73)^ab^	287 (39)^b^	0.006
Reaching its conspecifics /success of the test (point 4)	*Percentage of success*	25^a^	26.6^a^	13.3^a^	0.15
	*Latency*	265 (77)^a^	271 (71)^a^	289 (36)^a^	0.16

Different superscript letters within a row indicate significant differences between treatments (***P*** < 0.05).

### Novel object (NO) test

All data related to novel object (NO) observations are described in Table 9. As the test progressed, the birds increasingly approached and pecked the NO (*P* < 0.001). There were no significant differences among the treatments in the latency of the first bird to approach or peck the novel object on D13. There was a treatment effect regarding the latency to peck and to approach the NO at D27 (*P* = 0.01 and 0.04 respectively) but not at D13 (*P* = 0.07 and 0.24 respectively). The RDB broilers showed shorter latencies for approaching and peck the NO than RoCV broilers on D27, whereas no significant differences were found between RDB and RoE+ broilers, or between RoE+ and RoCV broilers for this parameter. Regardless of age, there was a treatment effect on the number of birds approaching and pecking the NO (*P* < 0.001). More RDB broilers were observed approaching and pecking the NO than RoE+ and RoCV broilers, with no differences between RoE+ and RoCV broilers, except on D27 when more RoE+ broilers approached the NO than RoCV broilers.

**Table 9:** Mean (± standard deviation) latency values in seconds and number of broilers to approach and peck the novel object on D13 and D27. GLMM with negative binomial distribution analyzed approach and pecking of the novel object. Latency values were compared using Kruskal-Wallis tests. RDB: Redbro broilers reared indoors with a limited enriched environment. RoCV: Ross 308 broilers with access to a covered veranda from 14 days of age. RoE+: Ross 308 broilers reared indoors with a complex enriched environment.

Variables	Age (A)		Treatments (T)			Significance of effects (*P*)					
			RDB	RoE+	RoCV	*T*			*A*		*TxA*
Latency before approaching the novel object		*D13*	141 (23)^a^	175 (57)^a^	209 (74)^a^	0.24					
		*D27*	8 (9)^a^	134 (112)^ab^	243 (187)^b^	0.04					
Latency before pecking the novel object		*D13*	287 (97)^a^	495 (120)^a^	524 (151)^a^	0.07					
		*D27*	33 (29)^a^	397 (157)^ab^	565 (70)^b^	0.01					
Number of broilers approaching the novel object		*D13*	10.1 (9.3)^a^	1.3 (1.8)^b^	0.5 (0.9)^b^	< 0.001		< 0.001			0.03
		*D27*	21.6 (8.4)^a^	4.1 (5.2)^b^	1.1 (1.9)^c^						
Number of broilers pecking the novel object		*D13*	2.0 (3.1)^a^	0.1 (0.3)^b^	0.0 (0.1)^b^	< 0.001	< 0.001			0.58	
		*D27*	6.1 (3.3)^a^	0.7 (1.6)^b^	0.1 (0.3)^b^						

Different superscript letters within a row indicate significant differences between treatments (***P*** < 0.05), as determined by post hoc tests.

## Discussion

The aim of this study was to compare the effects of three rearing systems, combining different levers for improving bird welfare (slower-growing genetic strain, indoor environmental enrichments, or access to a covered veranda). The slower growing birds had lower gait impairment, footpad dermatitis scores and better results to the detour and novel object tests; the indoor enrichments stimulated dustbathing behaviors; the covered veranda encouraged active behaviors such as running, foraging and play in the covered veranda.

### Covered veranda use

The RoCV broilers used the covered verandas all along the period, with their rapid growth rate not appearing to be an obstacle. In accordance with previous studies on covered veranda (Bergmann et al., 2017; Mocz et al., 2025) or free-range (Riber et al., 2018; Collet et al., 2024), they increasingly used the covered verandas as they got older, with a peak on the last day of the rearing period with over around 28% of the broilers recorded in the covered veranda. This is in line with the hypothesis that cautiousness played a role during the first days of access to a unknown environment (Bergmann et al., 2017).

The broilers used the covered veranda more when the temperature was between 18°C and 25°C (in accordance with the thermoneutral zone of chickens, which is generally between 16°C and 26°C (Lu et al., 2017)). Using the temperature humidity index (THI) formulation used in Kim et al. (2025), the preferred conditions in the present study are precisely categorized as thermally favorable conditions (THI <70) but also inducing potential mild heat stress (THI between 70 and 75) (Lahiry et al., 2025; Kim et al., 2025). On the contrary, the lowest number of birds observed in the covered veranda was recorded when environmental conditions ranged from those inducing mild heat stress to those classified within the danger category for heat stress (THI = 76–81). Nevertheless, thermal comfort cannot be fully explained by temperature and humidity alone, as air velocity has been shown to play a major role in mitigating heat stress and in broiler behaviors (Akter et al., 2022a, 2022b).

### Enrichments use by RoE+ broilers

The platforms were used more for perching than the linear perches, in line with previous studies showing that platforms are more suitable for fast-growing broiler chicken, as they are easier to access and heavier birds may have fewer balancing difficulties when resting on platforms (Riber et al., 2018). The platforms were used from the beginning of the rearing period, whereas the linear perches started to be used one week later. Platform ramps were not only used as an access to the top of the platform but also as a surface on which to perch/rest. However, the RoE+ broilers were observed lying on the top of the platform more frequently than on its ramps; this is in accordance with previous studies and in relation with the birds’ tendency to prefer horizontal rather than angled perches (Bailie et al., 2018; May et al., 2024).

### Behaviors

***Positive effects and limits of the indoor enrichments.*** Broilers from the RoE+ treatment exhibited more dustbathing behavior than birds from the other treatments, which may indicate a positive welfare state (Papageorgiou, 2023). According to Holt et al. (2023), broilers preferred fresh, fine-grained materials for dustbathing. However, the litter in the RoE+ pens did not meet these characteristics and did not differ from that of the other treatment pens. Although broilers in the covered veranda foraged more, likely due to the higher litter quality, they dustbathed very little. This could suggest that litter quality did not play a major role in the dustbathing behavior observed in this study, but the provision of indoor environmental enrichment did, in accordance with previous studies (Vasdal et al., 2019; Lourenco da Silva et al., 2021).

RoE+ broilers exhibited levels of activity (walking, running, and play behaviors) similar to those of RDB broilers, suggesting that this complex and enriched environment may have partially compensated for the effects of their faster growth rate, as environmental enrichment is known to positively influence activity levels in broilers (Riber et al., 2018; Vasdal et al., 2019; Guinebretiere et al., 2025). Although, the higher number of lying birds in the RoE+ treatment than in the RDB treatment may mitigate this hypothesis.

***Positive effects of the covered veranda*.** Ross 308 broilers observed in the covered verandas were more active than Ross 308 or Redbro broilers observed indoors, exhibiting more walking and running behaviors, whereas there were more lying birds indoors than in the covered verandas. This is consistent with previous studies reporting a higher activity in outdoor runs or covered verandas and more birds lying down indoors (Weeks et al., 1994; Ruis et al., 2004; Bergmann et al., 2017; Mocz et al., 2025). Similarly, more spontaneous sparring games and frolicking behaviors were observed in the covered verandas than indoors, where no difference was observed among treatments. Although genetics may influence play behavior, with faster-growing chickens less likely to express play behaviors than slower-growing breeds under the same conditions (Rayner, 2020; Baxter et al., 2021), in our study the rearing environment (the covered veranda) played a more prominent role in the expression of play behaviors than the breed itself. One explanation is the additional space available in the covered veranda; indeed, this is reported to stimulate play behavior among young broilers (Baxter et al., 2019; Liu et al., 2020, 2021), and as a higher stocking density may negatively affect birds’ locomotion (Bach et al., 2019; EFSA, 2023). In our study, the play, walking and running behaviors observed in the covered verandas did not appear to be related to the stocking density, as the number of birds in the covered verandas was higher than indoors in the RoCV pens at the frequentation peak. However, the enriched outdoor environment offered by the covered verandas, with their sensory stimulations (fresh air, odors, temperature, direct sunlight), may play a major role in the stimulation of active and play behaviors, that are positive welfare indicators (Papageorgiou et al., 2023), among broilers. These findings confirm that access to an outdoor or outdoor-like environment stimulates active behaviors in broilers, even in fast-growing strains, and suggest that fast-growing broiler chickens in a stimulating environment may be more active than slower-growing broilers kept indoors (here, Redbro broilers).

The broilers in the covered verandas also foraged more than broilers indoors, while no difference was revealed between the three treatments indoors. This may be explained by the provision of a different type of litter in the covered verandas compared with the indoor area. Another hypothesis concerns the higher quality of the litter in the covered veranda (minimally degraded; personal observations), which may have stimulated foraging behavior. Indeed, foraging behaviors are stimulated by the provision of fresh litter materials (Holt et al., 2023). In the indoor pens, litter quality was consistently suboptimal across all three treatments. Besides litter quality, foraging behavior in covered verandas may also have been stimulated by higher outdoor light intensity, as a positive relationship is known to exist between light intensity and foraging behavior (Alvino et al., 2009).

### Leg health and walking ability

At 32 days of age, RDB broilers showed fewer gait issues than both RoE+ and RoCV treatments, which may be related to differences in genetics or enrichments. However, there is a scientific consensus on lower walking ability issues in faster-growing strains (Baxter et al., 2021; Guinebretière et al., 2024; Riber and Wurtz, 2024), whereas previous studies have reported no or positive effects of indoor enrichments on walking ability (Kaukonen et al., 2017; Bailie et al., 2018; Mocz et al., 2022). Thus, this difference in gait issues between RDB broilers and both Ross 308 treatments is more likely due to growth rate.

At this age, RDB broilers also exhibited fewer footpad dermatitis lesions than RoE+ and RoCV birds, consistent with the generally poorer leg health reported in faster-growing strains, although footpad dermatitis is also strongly influenced by litter quality (Haslam et al., 2006; EFSA, 2023; Riber and Wurtz, 2024).

When broilers were compared at similar body weights, the difference in walking ability and footpad dermatitis remained between RDB and RoE+ birds but disappeared between RDB and RoCV birds. While access to a covered veranda does not appear, as in previous studies, to directly affect walking ability (Ruis et al., 2004; Mocz et al., 2025), this convergence between RDB and RoCV at similar body weights could suggest a positive effect of the veranda on walking ability and footpad dermatitis. For footpad dermatitis specifically, the improvement in RoCV birds reflects their regular access to better quality litter in the covered veranda, as the indoor litter quality did not differ between treatments, rather than a strain-related effect.

At 32 days of age, RoE+ broilers exhibited more hock burns than both RDB and RoCV birds, with no difference between the latter two, a pattern similar to that observed for walking ability and footpad dermatitis. However, when broilers were compared at similar body weights, this pattern reversed: Redbro broilers exhibited more hock burns than both Ross 308 treatments. This result cannot be explained by differences in walking ability, as RoCV birds had comparable gait scores and RoE+ birds had poorer ones. It may instead reflect the older age of Redbro broilers at a given body weight. Since hock burns develop later than footpad dermatitis (Kjaer et al., 2006), this longer exposure to litter may have increased their prevalence. Within the Ross 308 treatments, access to a covered veranda was associated with fewer hock burns in RoCV than in RoE+ birds, consistent with previous findings on Redbro broilers in covered verandas (Mocz et al., 2025). This likely reflects the higher activity levels of RoCV birds in the veranda, rather than litter quality alone. Indeed, although hock burns have multifactorial causes, including litter quality and genetic traits (Ask, 2010; EFSA, 2023; Riber and Wurtz, 2024), they are also considered indicators of prolonged lying behavior, itself a marker of inactivity (Haslam et al., 2006; de Jong et al., 2016).

### Novel object test

RDB broilers approached and pecked the novel object more than both Ross 308 treatments. Three main hypotheses can be proposed. First, Redbro broilers are less scared of an unknown object (trait of this strain). Secondly, they may have been more mobile due to their better gait, which could have facilitated interactions with the novel object, as previous studies have shown that the outcomes of fear tests requiring birds to move can be influenced by gait score (Vasdal et al., 2018; Rayner, 2020). However, the differences observed at D13, when birds are not expected to experience mobility issues, may not support this hypothesis. Thirdly, the barren rearing environment of the RDB broilers may have enhanced their motivation to interact with the novel object, which was a new stimulus.

RoE+ broilers also approached the novel object significantly more than RoCV broilers on D27. This may indicate that the stimuli provided by the covered veranda mitigated the motivation of RoCV birds to interact with the novel object, or that the indoor enrichments stimulated the curiosity of RoE+ birds. Indeed, previous studies have shown that birds reared with indoor enrichments may be more interested/less fearful in a novel object or unfamiliar human test (Guinebretiere et al., 2025), and a more complex rearing environment is known to decrease poultry fear level (Riber et al., 2018).

### Detour test

Fewer than one third of the birds passed the detour test successfully by reaching their conspecifics and the worms at point 4, with no treatment effect observed. This success rate is lower than that reported in previous studies realized with younger animals, such as Guinebretière et al. (2025), in which approximately 47% of 21–22-day-old Redbro broilers (with and without enrichments) succeeded, or Wichman et al. (2007), where 44% of 4-day-old Lohmann Selected Leghorn chicks completed the detour test. The detour test is a method used to assess poultry’s spatial abilities and their capacity to adapt to a novel situation (Ferreira et al., 2024). The present study outcomes may reflect low motivation, as most birds remained crouched down or moved around within the test arena while foraging during the test. Because the test was conducted within the rearing pens, the tested broilers could hear their conspecifics all around them, even if they could only see them through the fence at point 4. This may have reduced social motivation to reach the goal, as social isolation was only partial.

Similarly, to the novel object test, Redbro broilers were globally better in the detour test than Ross 308 broilers, whereas RoE+ treatment had better result in the detour test than RoCV. It can be hypothesized that access to an outdoor-like environment in RoCV broilers may have influenced their abilities or motivation in the detour test. Ferreira et al. (2024) showed that laying hens making greater use of the outdoor range (“high rangers”) were slower to learn the detour task than birds using the range less (“low rangers”). Moreover, the authors reported a reduction in social motivation following range access. Our results may similarly indicate a relationship between outdoor access and detour test success.

### Animal performance

As expected, Redbro broilers (intermediate-growing strain) were lighter than Ross 308 broilers from both treatments at the same age from D14. Consequently, they consumed more feed and water to reach the same final body weight than Ross 308 broilers, a strain which has been selected to prioritize rapid growth rates with a high feed efficiency (Tallentire et al., 2016). Otherwise, the relatively low mortality was not affected by the treatments.

Although no significant difference in daily feed consumption was noted between both Ross 308 treatments, the RoE+ broilers drank more water daily (from 24 days of age) than RoCV broilers. There is a strong relationship between feed and water consumption, and broiler water consumption is affected by environmental temperature (Fairchild and Ritz, 2015). We can hypothesize that RoCV broilers were exposed to cooler conditions due to access to the covered veranda than RoE+ broilers, which remained fully confined.

The two Ross 308 treatments’ birds did not differ in body weight, except on one day, preventing any strong conclusion. To the authors’ knowledge, the only study investigating access by broilers to covered verandas found no effect on body weight (Mocz et al., 2025), and there is no consensus on the impact of indoor enrichments on broiler body weights, probably because of the variability of strains and enrichments that have been tested (Ohara et al., 2015; de Jong et al., 2021; Mocz et al., 2022). More investigations are needed to understand how the enrichments influence body weights of broilers.

It should be noted that this study was designed to compare welfare outcomes among three distinct rearing systems, each combining different factors (slower growth rate, complex enriched indoor environment, covered veranda access). As such, our design does not allow to isolate the specific contribution of each individual factor to the welfare indicators assessed. Some inferences regarding the likely role of specific factors can nonetheless be drawn by comparison with previous studies that assessed these factors independently. However, such interpretations remain indirect and should be confirmed by future studies.

## Conclusion

In conclusion, the three rearing systems influenced different aspects of broiler welfare. The slower-growing strain indoor was associated with fewer leg-related issues, such as gait impairment and footpad dermatitis. The provision of indoor enrichments enabled fast-growing broilers to express species-specific behaviors and promoted more dustbathing in areas free of enrichments. Access to a covered veranda stimulated active behaviors such as running, foraging and play, while also improving footpad dermatitis in fast-growing broilers. Despite their higher growth rate, the Ross 308 broilers exhibited high levels of activity and positive welfare indicators in the covered verandas, indicating that even fast-growing genotypes, although typically less active, can use outdoor-like environments and express a wide range of behaviors when provided with stimulating conditions. Although indoor enrichments did not stimulate active behaviors in Ross 308 broilers to the same extent as access to a covered veranda, the enrichments were well used, and the high occurrence of dustbathing nevertheless reflected positive welfare. Our study exhibits that several pathways currently exist to improve the welfare of standard broiler chicken production.

## Declaration of AI and AI-assisted technologies in the writing process

During the preparation of this work, the authors wrote the original manuscript and used *ChatGPT* solely to check and refine the wording of specific sentences for readability and grammar. After using this tool, the content was reviewed and edited by the authors and a professional translator. The authors take full responsibility for the content of the publication.

## Author Contributions

Frédérique Mocz contributed to the conception and design of the experiment, participated in the acquisition of the data, and performed the analysis and interpretation of the outcomes. She wrote the manuscript and revised it following comments from co-authors and journal reviewers. She reviewed and approved the final version of the manuscript and agrees to be accountable for all aspects of the work. Camille Boulangé participated in the acquisition of the data, the analysis and interpretation of the outcomes. She reviewed the different versions of the manuscript and approved the final version of the manuscript and agrees to be accountable for all aspects of the work. Jean-Philippe Moysan contributed to the conception and design of the experiment, participated in the acquisition of the data and interpretation of the outcomes. He critically reviewed the different versions of the manuscript and approved the final version of the manuscript and agrees to be accountable for all aspects of the work. Laura Warin contributed to the conception and design of the experiment, including discussions on the experimental protocol, and participated in the interpretation of the results. She reviewed and approved the final version of the manuscript and agrees to be accountable for all aspects of the work. Alassane Keita contributed to the conception and design of the experiment. He critically reviewed the different versions of the manuscript and approved the final version of the manuscript and agrees to be accountable for all aspects of the work. Cécile Arnould contributed to the interpretation of data. She critically reviewed the manuscript and provided substantive intellectual input. She reviewed and approved the final version of the manuscript and agrees to be accountable for all aspects of the work. Bertrand Méda contributed to the conception and design of the experiment and participated in the interpretation of the results. He critically reviewed the different versions of the manuscript and provided substantive intellectual input. He reviewed the different versions of the manuscript and approved the final version of the manuscript and agrees to be accountable for all aspects of the work. Elisabeth Le Bihan-Duval contributed to the conception and design of the experiment and participated in the interpretation of the results. She critically reviewed the different versions of the manuscript and provided substantive intellectual input. She reviewed and approved the final version of the manuscript and agrees to be accountable for all aspects of the work. Maryse Guinebretiére contributed to the conception and design of the experiment, participated in the acquisition of the data, and interpretation of the outcomes. She critically reviewed the different versions of the manuscript and provided substantive intellectual input. She reviewed and approved the final version of the manuscript and agrees to be accountable for all aspects of the work.

## Disclosures

The authors declare that they have no known competing financial interests or personal relationships that could have appeared to influence the work reported in this paper.

## Footnotes

Supplementary material associated with this article can be found, in the online version, at doi:10.1016/j.psj.2026.107950.
