# Supplementary material for: Slower growth, indoor environmental enrichment, or covered veranda access: A comparison of three rearing systems for broiler chicken welfare and behavior
Source: Poult Sci. 2026 Oct 11;105(12):107950. doi: 10.1016/j.psj.2026.107950 (PMC13721923; doi:10.1016/j.psj.2026.107950)
Supplement: Supplementary file 1 [file mmc1.docx]

# Supplementary Data

**Table A1.** Mean (± standard deviation) of the standardized occurrence rates of each behavior observed in the four pens, and percentages of birds lying down, of RDB, RoE+, RoCV indoors (RoCV-I) and RoCV in the covered veranda (RoCV-CV) and comparisons at similar body weights: 675 g (= Ross 308 on D18 vs. RDB on D20); 800 g (= Ross 308 on D20 vs. RDB on D22); 950 g (= Ross 308 on D22 vs. RDB on D26); and 1,200 g (Ross 308 on D26 vs. RDB on D29). RDB: Redbro broilers reared indoors with a limited enriched environment. RoCV: Ross 308 broilers with access to a covered veranda from 14 days of age. RoE+: Ross 308 broilers reared indoors with a complex enriched environment.

| **Behavior** | **BW Comparisons** | **Location** | | | | Significance of effects (***P***) | | |
| --- | --- | --- | --- | --- | --- | --- | --- | --- |
|  |  | **RDB** | **RoE+** | **RoCV-I** | **RoCV-CV** |  |  |  |
| **Walking^1^** | ***675 g*** | 37.1 (27.6)^b^ | 35.7 (15.6)^b^ | 26.3 (14.2)^b^ | 394.8 (175.7)^a^ | **<0.001** | | |
|  | ***800 g*** | 49 (20.9)^b^ | 24 (11.9)^b^ | 38.5 (57.5)^b^ | 256.7 (160.4)^a^ | **<0.001** | | |
|  | ***950 g*** | 67.9 (34.2)^b^ | 20.7 (10.5)^b^ | 30.9 (21.4)^b^ | 447.8 (202.1)^a^ | **<0.001** | | |
|  | ***1,200 g*** | 20.4 (5.2)^b^ | 23.8 (11.7)^b^ | 30.1 (16.6)^b^ | 306.5 (102.3)^a^ | **<0.001** | | |
| **Running^1^** | ***675 g*** | 4.3 (5.9)^b^ | 1.3 (1.7)^b^ | 5.8 (8.4)^b^ | 77.6 (84.8)^a^ | **0.001** | | |
|  | ***800 g*** | 7.2 (11.4)^b^ | 2.6 (4.4)^b^ | 12.5 (21.3)^b^ | 109.6 (110.3)^a^ | **<0.001** | | |
|  | ***950 g*** | 11.0 (21.7)^b^ | 1.3 (1.3)^b^ | 2.1 (2.9)^b^ | 51.5 (47.3)^a^ | **<0.001** | | |
|  | ***1,200 g*** | 5.0 (3.4)^b^ | 0.2 (0.5)^b^ | 2.5 (3)^b^ | 53.2 (30.3)^a^ | **<0.001** | | |
| Foraging^1^ | ***675 g*** | 10.2 (10)^b^ | 18.7 (13.3)^b^ | 8.7 (6.6)^b^ | 337.9 (128.1)^a^ | **<0.001** | | |
|  | ***800 g*** | 13.4 (8.3)^b^ | 11.1 (6.8)^b^ | 21.6 (39)^b^ | 402.1 (140.3)^a^ | **<0.001** | | |
|  | ***950 g*** | 17.0 (24.3)^b^ | 6.2 (6.5)^b^ | 10.7 (18.7)^b^ | 385.2 (147.6)^a^ | **<0.001** | | |
|  | ***1,200 g*** | 1.7 (2)^b^ | 4.5 (4.6)^b^ | 3.4 (3)^b^ | 209.0 (23.8)^a^ | **<0.001** | | |
| Dustbathing^1^ | ***675 g*** | 23.8 (26.5)^ab^ | 25.6 (23.4)^a^ | 15.1 (13.8)^ab^ | 0.0 (0.0)^b^ |  | **0.04** |  |
|  | ***800 g*** | 3.6 (5)^a^ | 40.0 (38.9)^a^ | 33.8 (49.7)^a^ | 0.0(0.0)^a^ |  | **0.02** |  |
|  | ***950 g*** | 5.4 (5.7)^b^ | 58.4 (41.1)^a^ | 41.0 (19.5)^a^ | 0.0(0.0)^b^ |  | **<0.001** |  |
|  | ***1,200 g*** | 0.2 (0.5)^b^ | 15.4 (14.2)^a^ | 4.1 (6.8)^b^ | 0.0(0.0)^b^ |  | **0.001** |  |
| Sparring games^1^ | ***675 g*** | 1.3 (2.6)^b^ | 1.2 (2.2)^b^ | 6.4 (11.2)^b^ | 162.8 (81.3)^a^ |  | **<0.001** |  |
|  | ***800 g*** | 5.7 (8.3)^b^ | 2.0 (3.4)^b^ | 19.1 (43.6)^b^ | 226.1 (149)^a^ |  | **<0.001** |  |
|  | ***950 g*** | 3.8 (8.2)^b^ | 0.0(0.0)^b^ | 0.0(0.0)^b^ | 189.2 (76.3)^a^ |  | **<0.001** |  |
|  | ***1,200 g*** | 1.1 (1.1)^b^ | 0.3 (0.9)^b^ | 0.2 (0.6)^b^ | 80.9 (39.7)^a^ |  | **<0.001** |  |
| **Frolicking^1^** | ***675 g*** | 10.2 (7)^b^ | 12.1 (9.5)^b^ | 15.6 (16.5)^b^ | 444 (268.5)^a^ |  | **<0.001** |  |
|  | ***800 g*** | 24.5 (29.4)^b^ | 11.7 (10)^b^ | 22.6 (35.3)^b^ | 424.5 (236.1)^a^ |  | **<0.001** |  |
|  | ***950 g*** | 17.9 (15.7)^b^ | 4.3 (3.2)^b^ | 7.2 (6.9)^b^ | 371.8 (191.4)^a^ |  | **<0.001** |  |
|  | ***1,200 g*** | 4.3 (5.7)^b^ | 3.3 (2.3)^b^ | 6.3 (4.5)^b^ | 240.9 (138.9)^a^ |  | **<0.001** |  |
| Percentage of birds lying down | ***675 g*** | 88.9 (7)^a^ | 93.6 (5.2)^a^ | 96.6 (4.4)^a^ | 32.0 (34.1)^b^ |  | **<0.001** |  |
|  | ***800 g*** | 85.3 (14.2)^a^ | 94.9 (13.4)^a^ | 91.3 (6.3)^a^ | 31.2 (19.8)^b^ |  | **<0.001** |  |
|  | ***950 g*** | 79.6 (20.6)^b^ | 96.0 (4.3)^a^ | 96.8 (4.1)^a^ | 23.1 (22.3)^c^ |  | **<0.001** |  |
|  | ***1,200 g*** | 72.6 (22.4)^a^ | 94.4 (24)^a^ | 89.6 (6.4)^a^ | 24.7 (19.3)^b^ |  | **<0.001** |  |

^1^ number of occurrences of behavior per 100 broilers per 3-min observation period

Different superscript letters within a row indicate significant differences between treatments (***P*** < 0.05), as determined by post hoc tests.
